# Effects of a skin type diversity seminar on undergraduate medical students’ self-assessed competence in managing skin diseases in patients with skin of color

**DOI:** 10.1186/s12909-024-05828-x

**Published:** 2024-08-07

**Authors:** Finn Abeck, Ines Heinen, Rachel Sommer, Christine Blome, Martin Härter, Matthias Augustin, Stefan W. Schneider, Inga Hansen-Abeck, Nina Booken

**Affiliations:** 1 Department of Dermatology and Venereology, University Medical Center Hamburg-Eppendorf, Hamburg, Germany; 2 Department of Medical Psychology, University Medical Center Hamburg-Eppendorf, Hamburg, Germany; 3 Institute for Health Services Research in Dermatology and Nursing (IVDP), University Medical Center Hamburg-Eppendorf, Hamburg, Germany

**Keywords:** Skin type diversity, Skin of colour, Dermatology, Dermatology education, Medical students, Undergraduate training

## Abstract

**Background:**

Skin diseases in patients with skin of colour (Fitzpatrick skin types IV to VI) are underrepresented in dermatology training, which may lead to lower quality of care for these patients. To address this underrepresentation in medical education, a newly developed seminar on skin type diversity using an interactive teaching method was implemented in an undergraduate medical curriculum. This study examined the effects of a seminar on the self-assessed competence of medical students in managing skin conditions in patients with skin of colour.

**Methods:**

A questionnaire survey was conducted among fourth-year undergraduate medical students at the University of Hamburg (Germany) between October 2023 and February 2024. Students’ self-assessed competence was compared before and after the obligatory seminar (pre- and post-design).

**Results:**

In total, 158 students participated in the survey. After the seminar, knowledge of the presentation of skin diseases in patients with skin of colour and the associated psychological burden, differences in the incidence of skin diseases in different skin types, and the ability to diagnose skin diseases in darker skin types increased. Most participants stated that they wanted to attend more courses on this topic.

**Discussion:**

Appropriate courses for medical students can improve their competence in managing different skin diseases in patients with skin of colour. In the future, more attention should be paid to teaching the diversity of skin types in dermatology education.

## Background

Currently, physicians are faced with an increasingly diverse patient population [1]. In the field of dermatology, diversity includes treating patients with different skin types. According to Fitzpatrick, six different skin types can be distinguished based on sunburn and tanning behaviour, ranging from skin type I (very light skin) to VI (dark brown to black skin) [2]. People with skin of colour (SoC) can be characterised as having skin types IV to VI [3].

Studies in the United Kingdom and United States have demonstrated a difference in the quality of medical care between patients with fair skin and those with SoC. [4, 5] One reason for this may be the underrepresentation of dermatoses in darker skin types in educational literature, which indicates that students and dermatologists are not adequately trained [5]. A lack of knowledge of the presentation of skin conditions in people with darker skin tones may contribute to delays in the diagnosis and treatment of patients. As this can put patients at risk, better training on diagnosing skin diseases in patients with SoC is needed [6].

According to a survey in Denmark, medical students and junior doctors feel less confident in diagnosing skin diseases in people with SoC than when diagnosing them in people with lighter skin types [7]. Darker skin types are also underrepresented in dermatology training in Germany, as textbooks focus on skin types I to III according to Fitzpatrick [8]. This imbalance could lead to a lower standard of medical care for people with SoC in Germany. Considering the increasing diversity of skin types in the population, this problem needs to be addressed by adapting academic teaching both in the undergraduate medical curriculum and specialist training [8]. In addition to the purely medical aspects, the psychological burden of skin diseases in people with SoC and light skin is enormous and should be considered by physicians [9].

The limited representation of skin type diversity in dermatology education weakens the ability of future physicians to diagnose and treat patients with skin of color [10]. According to a recently published study from the United States, students cite inadequate curriculum coverage as a major reason for their lack of confidence in diagnosing skin disease in patients with SoC [11]. Future research is needed to identify the most effective ways to improve education about skin type diversity in medical education and to understand how these approaches can help build the skills necessary to provide culturally competent care [12]. To our knowledge, few studies have been published on teaching skin type diversity in medical education curricula, none of which have been conducted in Germany [10, 11, 13]. In particular, there is a lack of studies on programs that have been implemented as an obligatory part of the curriculum. To better align the medical curriculum with the increasing importance of the growing diversity of skin types in the patient population, studies on the effectiveness and student attitudes towards newly developed programs on this topic are of great importance. To counteract the underrepresentation of dermatoses of darker skin types in medical education in Germany, a newly developed seminar on skin type diversity was implemented in the undergraduate medical curriculum at Medical University Hamburg. This study investigated the effects of a seminar on medical students’ self-assessed competence in managing skin diseases in patients with SoC.

## Methods

A pre- and post-questionnaire survey of medical students at Medical University Hamburg was conducted between October 2023 and February 2024. A seminar was conducted in the fourth year of the 6-year medical curriculum [14]. Prior to study initiation, the study design was reviewed and approved by the local Psychological Ethics Committee of the Center for Psychosocial Medicine (LPEK-0688). The students were provided an information sheet regarding the study prior to the survey. Survey participation was voluntary and anonymous. The participants did not receive any compensation. Nonparticipation in the survey had no negative consequences for students. Informed consent was obtained from all participants.

The seminar was implemented as a mandatory part of the curriculum (group size of approximately 20 students each), with the participation of the Department of Dermatology, Department of Medical Psychology, and Institute for Health Services Research in Dermatology and Nursing. As part of the undergraduate curriculum at the University of Hamburg, students are required to attend at least 85% of mandatory courses. This indicates that an attendance rate of < 100% is possible, despite the seminar being compulsory.

In addition to the topic of skin diseases in people with SoC, the seminar covered psychological comorbidities in chronic skin diseases and stigmatisation. Because the latter two topics were not specifically related to people with SoC, they were excluded from this study. The seminar was scheduled for 90 min, with 45 min devoted to teaching skin-type diversity.

The topics covered in the seminar were based on the current literature on skin diseases in people with SoC [4, 5, 8, 9, 15]. This educational program used an interactive teaching method, including brainstorming (why is it important to know about skin diseases in darker skin tones? ) and a case-based scenario (patient with SoC who was misdiagnosed). In addition to presenting various dermatological conditions on different skin types (including psoriasis vulgaris, acne vulgaris, herpes zoster, malignant melanoma, and keloids) and comparing the prevalence of individual skin diseases on different skin types, [16] students were actively involved in class discussions on differential diagnosis and further diagnostic and therapeutic measures for the presented conditions. Furthermore, psychological burdens of people with SoC affected by skin diseases (including feelings that physicians do not know how to treat skin conditions in patients with SoC, [9] increased incidence of post-inflammatory hyperpigmentation, [17] and increased visibility of vitiligo lesions [18]) were discussed. At the time of the seminar, at least 75% of the dermatology lectures in the curriculum had already been conducted; therefore, a certain level of dermatological knowledge among the students could be assumed.

A paper-based questionnaire was designed by the authors to assess the students’ self-assessed competence and attitude towards skin diseases in patients with SoC. The questionnaire was reviewed for comprehensibility and plausibility by experts in the field of dermatology and student surveys. Ratings were made on a Likert scale ranging from 1 to 6 (relevance/knowledge “very low” to “very high”). As there is no specific course on skin type diversity in the medical curriculum in Germany, no validated questionnaires existed. Therefore, we based the form and structure of the questionnaire on the regular teaching evaluations of the undergraduate medical curriculum of the University of Hamburg. Thus, the participants were already familiar with the survey method. The students completed the written questionnaire before and after the seminar. In addition to general student characteristics (age, sex, migration background, self-assessment as a person with SoC, current semester of study, and previous training/studies), the relevance of knowledge and knowledge of the presentation of dermatological conditions in people with SoC at the beginning and end of the seminar, the knowledge of the differences in the incidence of skin diseases according to skin type, the ability to make a dermatological diagnosis in patients with SoC, and the relevance of knowledge and knowledge of the psychosocial burden caused by skin diseases in people with SoC according to self-assessment were evaluated. In addition, at the end of the course, content and structure of the seminar were evaluated (Likert scale from 1 to 6; statement “not at all relevant” to statement “very relevant”).

Data were analysed using GraphPad Prism version 8 (GraphPad Software, San Diego, CA, USA) and IBM SPSS Statistics version 29. Paired sample *t-tests* were used to compare the pre- and post-seminar questionnaires on knowledge and skills. Statistical tests were one-tailed, and the α level of 5% was set at *p* < 0.0083 after Bonferroni adjustment for multiple testing. The tests were one-tailed because participation in the seminar was expected to lead to an increase in self-assessed competence and knowledge, as well as a higher rating of the relevance of the seminar content. A quasi-experimental pre–post design without a control group or randomisation was adopted, as the new seminar was introduced in parallel with all existing seminar groups of the curriculum in the winter semester of 2023.

## Results

Of the 184 students, 162 attended the seminar (88.0%). The voluntary survey participation rate was 97.5% (*n* = 158). Of the students, 64.3% were women (*n* = 101), and the mean age was 24.2 ± 2.8 years (range, 20–36 years). Germany was reported as the country of birth by 87.9% (*n* = 138) of the students, and 10.5% (*n* = 16) identified themselves as people with SoC. Of the students, 83.4% (*n* = 128) were in their 7th semester, and 46.7% (*n* = 71) stated that they had completed vocational training or a course of study before starting their medical studies (Table 1).

**Table 1:** General student characteristics

	Students (*n* = 158)
**Age**	
Mean value (SD)	24,2 (2,8)
Range	20–36
**Sex**, ***n*(%)**	
Female	101 (64,3)
Male	55 (35,0)
Diverse	1 (0,6)
**Self-assessment as a person with SoC**, ***n*(%)**	16 (10,5%)
**Semester**, ***n*(%)**	
Six	8 (5,2)
Seven	128 (83,7)
Eight	6 (3,9)
Nine	11 (7,2)
**Previous training/studies**, ***n*(%)**	71 (46,7)

SD: standard deviation; SoC: skin of colour

**Fig. 1:**
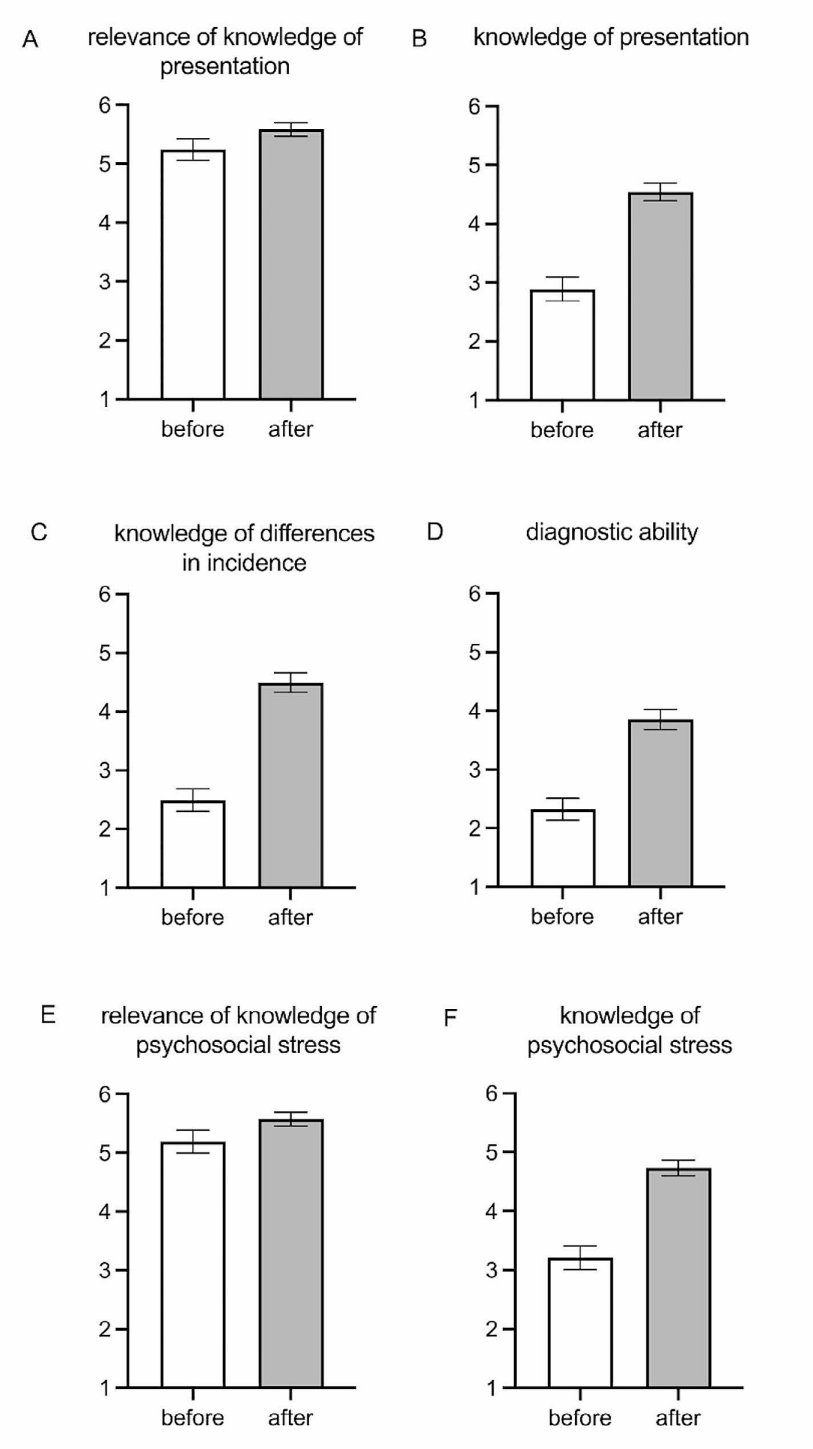
Means and 95% confidence interval of the students’ self-assessment (*n = 158*); survey before and after the seminar. **A**) Assessment of the relevance of knowledge of the presentation of dermatological conditions in people with skin of colour (SoC). **B**) Knowledge of the presentation of dermatological conditions in people with SoC. **C**) Knowledge of differences in the incidence of skin diseases according to different skin types. **D**) Assessment of own ability to diagnose skin diseases in people with SoC. **E**) Assessment of the relevance of knowledge of psychosocial stress caused by skin diseases in people with SoC. **F**) Knowledge of psychosocial stress caused by skin diseases in people with SoC

Paired-sample *t-tests* were used to compare students’ self-assessment of the six items before and after the seminar. The relevance of knowledge of the presentation of skin diseases in patients with SoC was rated on average at 5.2 ± 1.2 before the seminar (Likert scale from 1 “very low relevance” to 6 “very high relevance”). The students’ self-assessed knowledge of the presentations of skin diseases in people with SoC averaged 2.9 ± 1.3 (Likert scale from 1 “very low knowledge” to 6 “very high knowledge”). After the seminar, the students rated both relevance (5.6 ± 0.7; *t*(157) = − 14.610, *p* < 0.001) and knowledge (4.5 ± 0.9; *t*(157) = − 4.158, *p* < 0.001) as significantly higher than before the seminar (Fig. 1A and B).

Regarding the knowledge of differences in the incidence of skin diseases according to skin type, a significant increase in knowledge was observed after the seminar according to self-assessment (2.5 ± 1.2 vs. 4.5 ± 1.1; *t*(157) = − 18.007, *p* < 0.001; Likert scale from 1 “very low knowledge” to 6 “very high knowledge”; Fig. 1C). The students’ self-assessed ability to make a dermatologic diagnosis in people with SoC increased significantly from 2.3 ± 1.2 (Likert scale from 1 “very low ability” to 6 “very high ability”) before the seminar to 3.9 ± 1.1 after the seminar (*t*(157) = − 14.645, *p* < 0.001) (Fig. 1D).

The relevance of knowledge of psychosocial burden caused by skin conditions in people with SoC was rated by the students before the seminar with an average of 5.2 ± 1.2 (Likert scale from 1 “very low relevance” to 6 “very high relevance”). The students’ self-assessed knowledge of psychosocial stress was on average 3.2 ± 1.3 (scale from 1 “very little knowledge” to 6 “very high knowledge”). After completing the seminar, the students rated both relevance (5.6 ± 0.8; *t*(157) = − 15.212, *p* < 0.001) and knowledge of psychosocial stress (4.7 ± 0.9; *t*(157) = − 4.441, *p* < 0.001) caused by skin diseases in people with SoC significantly higher than before the seminar (Fig. 1E and F).

The results of the paired t-tests are shown in Table 2. The effect sizes of Cohen’s d > 0.8 indicate a large effect.

**Table 2:** Descriptive data (mean [M], standard deviation [SD], median [Md], percentile 25 [Q1], and percentile 75 [Q3]) as well as information on the paired-sample *t-tests* (mean difference [M diff.], 95% confidence interval of the difference [95% CI]) on the self-assessments of the students (*n = 158*) before and after seminar participation, including Cohen’s d for effect size

	Before					After					M Diff	95% CI				One-sided *p*	Cohen’s d
	M	SD	Md	Q1	Q3	M	SD	Md	Q1	Q3		Lower value	Upper value				
**A)** How do you rate the relevance of knowledge of the presentation of dermatological conditions in people with SoC?	5,2	1,2	6,0	5,0	6,0	5,6	0,7	6,0	5,0	6,0	−0,3	−0,5		-0,2	<,001		1,05
**B)** How would you rate your knowledge of the presentation of dermatological conditions in people with SoC?	2,9	1,3	3,0	2,0	4,0	4,5	0,9	5,0	4,0	5,0	−1,7	−1,9		−1,4	<,001		1,43
**C)** How would you rate your knowledge of the differences in the incidence of dermatological conditions in people with SoC?	2,5	1,2	2,0	2,0	3,0	4,5	1,1	5,0	4,0	5,0	−2,0	−2,2		−1,8	<,001		1,40
**D)** How would you rate your ability to diagnose skin conditions in people with SoC?	2,3	1,2	2,0	1,0	3,0	3,9	1,1	4,0	3,0	5,0	−1,6	−1,7		−1,3	<,001		1,32
**E)** How do you rate the relevance of knowledge of psychosocial stress caused by skin conditions in people with SoC?	3,2	1,3	3,0	2,0	4,0	4,7	0,9	5,0	4,0	5,0	−1,5	−1,7		−1,3	<,001		1,26
**F)** How would you rate your knowledge of psychosocial stress caused by skin conditions in people with SoC?	5,2	1,2	6,0	5,0	6,0	5,6	0,8	6,0	5,0	6,0	−0,4	−0,6		−0,2	<,001		1,09

SoC, skin of colour

**Fig. 2:**
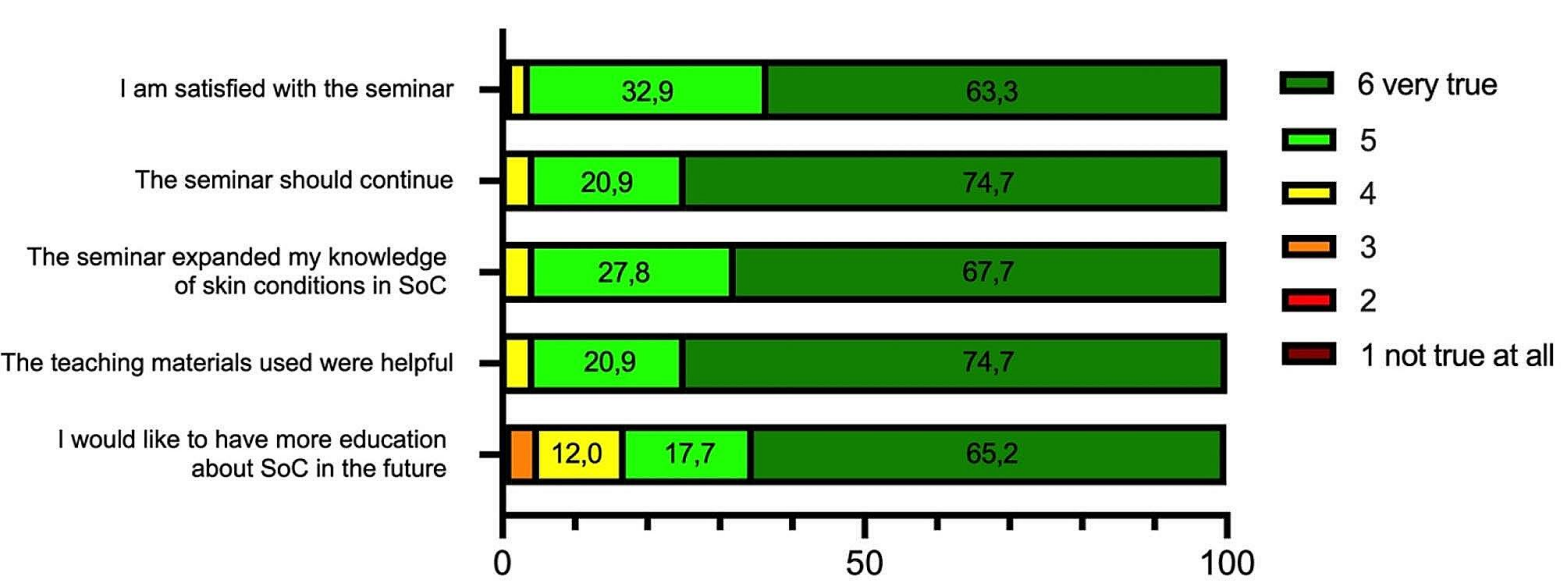
Students’ evaluation of the seminar (*n = 158*; 6-point Likert scale from 1 “not true at all” to 6 “very true”), showing the percentage distribution of responses

The majority of the students were satisfied with the seminar (96.2% selected 5 or 6 on a scale from 1 “not true at all” to 6 “very true”; *n* = 152) and stated that they had increased their knowledge on skin diseases in people with SoC (95.5% with a rating of 5 or 6 on the 6-point Likert scale; *n* = 151). Additionally, 82.9% of the respondents indicated that they would like to attend more courses on this topic in the future (rating 5 or 6; *n* = 131) (Fig. 2).

## Discussion

Recent research has highlighted the underrepresentation of skin of colour in medical school dermatology education and the limited diversity observed within the field [19]. This study investigated the effects of a newly designed seminar on skin diseases in patients with SoC on medical students’ self-assessed competence. To the best of our knowledge, this is the first study on this topic within the undergraduate medical curriculum in Germany. Many studies on dermatology education for medical students include only small groups of students with often < 25 participants [11], whereas this study involving 158 students is one of the larger studies, and the seminar was obligatory for all students.

Our results show that the self-assessed competence of medical students regarding skin diseases in people with SoC can be improved by offering appropriate courses. A significant increase in knowledge of the presentation of skin diseases in people with SoC and on differences in the incidence of skin diseases according to skin type was observed. The ability to make dermatological diagnoses of darker skin types also significantly improved according to students’ self-assessment. Owing to the increasing diversity of skin types, dermatological knowledge and skills regarding diagnosing skin diseases in people with SoC are of great importance to all medical students and physicians. Because many students are only exposed to dermatology as part of their medical curriculum, offering seminars on skin diseases in people with SoC during their studies may be important. Hence, most participants stated that more courses on this topic should be included in the medical curriculum. To the best of our knowledge, no comparable study involving medical students has been conducted to date in Germany. A recently published study assessed students’ ability to diagnose skin diseases in patients with SoC and their views on skin diversity education in the medical curriculum. 78.7% of participants felt that institutional learning resources for skin diversity education were inadequate. After the seminar, 87.2% of students expressed a lack of confidence in diagnosing skin diseases in patients with SoC, with most of them stating that this was due to inadequate curriculum coverage [11]. However, because it was a virtual seminar with voluntary participation, we believe our study offers relevant new insights beyond the existing literature. Another study from the United States also involving students showed that skin diseases were misdiagnosed more frequently in people with SoC than in those with fair skin types. However, this study did not include an intervention to improve competence [20]. In 2021, Mhlaba et al. evaluated the effects of a one-week curriculum on the management of skin diseases in people with SoC for dermatology residents in the USA. The authors showed that participants rated themselves as significantly more confident and able to treat dermatoses on darker skin after completing the seminar [21].

In addition to the acquisition of primary medical skills, our study results showed that the seminar also improved self-assessed knowledge of psychosocial stress caused by skin diseases in people with SoC. The potential negative impact of skin conditions on the psychosocial well-being of patients of all skin types is well known; people with skin diseases are more likely to have depressive symptoms, social isolation, loneliness, and a lower quality of life [22]. In a study conducted in 2022, which only included people with SoC, 57% of respondents reported an impact of the skin condition on their mental health. In addition, 46% reported that dermatologists do not have the necessary knowledge to treat skin conditions in darker skin types [9]. To the best of our knowledge, there is no existing program for medical students that links skin type diversity education to psychosocial stress. Sensitising prospective physicians to this topic and to the psychosocial burden of skin diseases in people with SoC during their medical studies could help reduce the discrepancy in the quality of medical care in the future. As the teaching time for dermatology is limited within the undergraduate medical curriculum, interdisciplinary teaching approaches can also be used to teach dermatological content [23]. The combination of dermatology and psychology presented here is a suitable approach because of the high psychosocial stress associated with many skin diseases.

The current literature calls for counteracting the underrepresentation of skin diseases in people with SoC in the context of education [4, 6, 8, 24]. This is also reflected in our subjective perception as teachers at the University of Hamburg, as we were frequently asked by students to focus more on skin diseases of people with darker skin types. The students’ awareness of this important topic in dermatology could have been raised through the media or by attending congresses. In addition, the underrepresentation of skin diseases in patients with SoC in national and international textbooks was addressed at the beginning of the seminar, and brainstorming was conducted about the importance of this topic. An educational program on skin type diversity fills a gap in the medical curriculum, which may explain the high level of student satisfaction observed in our study. However, the format of a targeted course on skin diseases in people with SoC is sometimes viewed critically, as this topic should not be titled as an additional area but should be integrated into the curriculum. Although our seminar was specifically designed to address skin conditions in people with SoC, it is a mandatory course on skin-type diversity in the undergraduate medical curriculum. A focused course on this topic may serves as a foundation upon which students can build as they progress through training. However, we plan to incorporate images of skin diseases in darker skin types more frequently into our lectures and seminars to make them topics for discussion. Therefore, we have revised the slide sets for all our dermatology courses to address the underrepresentation of skin diseases in darker skin types.

There is a shift from traditional teaching to student-centered approaches that actively engage students [25]. Our study suggests that the interactive teaching approach was effective in improving medical students’ self-assessed competence in managing skin diseases in patients with SoC. The interactive teaching method actively engages students and facilitates the exchange of ideas between students and teachers [26]. For students, interactivity can promote active learning and increase motivation and attention [27]. We believe that the interactive teaching approach used may be a reason for the high level of student satisfaction with the newly developed skin type diversity seminar. 12% of students were neutral on the question “I would like to have more education about SoC in the future”. The high workload that medical students already face in their curriculum may explain this finding. Additional courses may contribute to a crowded curriculum, leading to student dissatisfaction or even burnout [28].

This study has some limitations. First, response bias may be possible because of social desirability or the Hawthorne effect. This cannot be completely ruled out, especially in the case of the student–teacher relationship, as the students completed the questionnaire at the beginning and end of the seminar. Nevertheless, the survey was voluntary and anonymous, and non-participation had no negative consequences for the students. Second, knowledge and competence were assessed solely through self-assessment. However, the quasi-experimental pre–post design without a control group did not allow for causal conclusions as learning effects could have resulted from double questioning alone. Whether these corresponded to the acquired skills and in particular, whether they could be successfully applied in everyday clinical practice were not evaluated. The researcher-made questionnaire used in this study has not been validated, which may affect the validity and reliability of the data. Lastly, a final knowledge test as part of the study was not conducted, as the time required for this would have been at the expense of the seminar content. To determine whether the newly acquired knowledge can be applied in clinical practice, future studies should incorporate a knowledge test following seminar participation. As the survey was conducted directly after the seminar, no statements could be made on the possible long-term effects on students’ knowledge and competence regarding skin diseases in people with SoC. This should be investigated further in future studies.

## Conclusion

This study is the first to evaluate a newly designed seminar on skin type diversity in an undergraduate medical curriculum in Germany. After the seminar, competence regarding managing skin diseases in people with SoC significantly increased according to the students’ self-assessment. Developing continuing education programs for both students and dermatologists may help improve their competence in diagnosing skin diseases in people with SoC in the future. In addition, the discrepancy between the frequency of illustrations of light and dark skin types in textbooks needs to be reduced to integrate skin diseases of darker skin types into everyday learning environments.

## Data Availability

The data that support the findings of this study are available from the corresponding author upon reasonable request.

## Abbreviations

SoC: Skin of color

## References

[1] Jhutti-Johal J. Understanding and coping with diversity in healthcare. Health Care Anal. 2013;21(3):259–70. 23719755 10.1007/s10728-013-0249-0

[2] Gupta V, Sharma VK. Skin typing: Fitzpatrick grading and others. Clin Dermatol. 2019;37(5):430–6. 31896400 10.1016/j.clindermatol.2019.07.010

[3] Taylor SC, Cook-Bolden F. Defining skin of color. Cutis. 2002;69(6):435–7. 12078844

[4] Kamath P, Sundaram N, Morillo-Hernandez C, Barry F, James AJ. Visual racism in internet searches and dermatology textbooks. J Am Acad Dermatol. 2021;85(5):1348–9. 33130182 10.1016/j.jaad.2020.10.072

[5] Passby L. Melanin matters: ethnic diversity in dermatology textbooks. Med Teach. 2021;43(2):244–5. 32755423 10.1080/0142159X.2020.1802000

[6] Lester JC, Taylor SC, Chren MM. Under-representation of skin of colour in dermatology images: not just an educational issue. Br J Dermatol. 2019;180(6):1521–2. 31157429 10.1111/bjd.17608

[7] Ashur N, Sorensen J, Thomsen SF, Saunte DM, Thyssen JP, Norredam M. A survey of students’ and junior doctors’ confidence in diagnosing in skin of colour. Dan Med J. 2023;70(10). 37897391

[8] Gregersen DM, Elsner P. Ethnic diversity in German dermatology textbooks: does it exist and is it important? A mini review. JDDG: J Der Deutschen Dermatologischen Gesellschaft. 2021;19(11):1582–9. 10.1111/ddg.14543 34585822

[9] Cartwright MM, Kamen T, Desai SR. The Psychosocial Burden of skin disease and Dermatology Care insights among skin of Color consumers. J Drugs Dermatology: JDD. 2023;22(10):1027–33. 37801524 10.36849/JDD.7713

[10] Motta AJ, Thiede R, Stanescu C, Corral J. Constructing a culturally conscious Dermatology Image Collection for Undergraduate Medical Education. Acad Med. 2022;97(11S):S137. 10.1097/ACM.0000000000004866

[11] Arza A, Sejdiu Z, Viveiros M, James A, Weingarten M, Giordano C. Beyond the surface: unveiling gaps in medical education through eyes of diverse learners. Arch Dermatol Res. 2024;316(5):187. 38775979 10.1007/s00403-024-02963-9

[12] Murina A. Reply to letter to the editor, medical students’ ability to diagnose common dermatologic conditions in skin of color. J Am Acad Dermatol. 2020;83(6):e457. 32735969 10.1016/j.jaad.2020.07.103

[13] Okoro U, Chau TQ, Kawaoka J, Wong V, Qureshi AA. Skin of Color in Preclinical Medical Education: a cross-institutional comparison and a call to action. Cutis. 2021;108(4):204–9. 34847000 10.12788/cutis.0362

[14] Rheingans A, Soulos A, Mohr S, Meyer J, Guse AH. The Hamburg integrated medical degree program iMED. GMS J Med Educ. 2019;36(5):Doc52. 31815162 10.3205/zma001260 PMC6883244

[15] Onyekaba G, Taiwò Née Ademide Adelekun D, Lipoff JB. Skin of color representation in dermatology must be intentionally rectified. J Am Acad Dermatol. 2022;87(1):e43–4. 35339590 10.1016/j.jaad.2022.03.032

[16] Schmid-Grendelmeier P. Dermatosen auf dunkler Haut. hautnah dermatologie. 2022;38(1):28–33. 10.1007/s15012-021-6808-4

[17] Anvery N, Christensen RE, Dirr MA. Management of post-inflammatory hyperpigmentation in skin of color: a short review. J Cosmet Dermatol. 2022;21(5):1837–40. 35289059 10.1111/jocd.14916

[18] Ezzedine K, Grimes PE, Meurant JM, Seneschal J, Léauté-Labrèze C, Ballanger F, et al. Living with vitiligo: results from a national survey indicate differences between skin phototypes. Br J Dermatol. 2015;173(2):607–9. 25892476 10.1111/bjd.13839

[19] Mangion SE, Phan TA, Zagarella S, Cook D, Ganda K, Maibach HI. Medical school dermatology education: a scoping review. Clin Exp Dermatol. 2023;48(6):648–59. 36753386 10.1093/ced/llad052

[20] Fenton A, Elliott E, Shahbandi A, Ezenwa E, Morris C, McLawhorn J, et al. Medical students’ ability to diagnose common dermatologic conditions in skin of color. J Am Acad Dermatol. 2020;83(3):957–8. 32017947 10.1016/j.jaad.2019.12.078 PMC7447081

[21] Mhlaba JM, Pontes DS, Patterson SS, Kundu RV. Evaluation of a skin of Color Curriculum for Dermatology residents. J Drugs Dermatology: JDD. 2021;20(7):786–9. 34232004 10.36849/JDD.6193

[22] Yew YW, Kuan AHY, Ge L, Yap CW, Heng BH. Psychosocial impact of skin diseases: a population-based study. PLoS ONE. 2020;15(12):e0244765. 33382864 10.1371/journal.pone.0244765 PMC7775076

[23] Bernges F, Zielbauer S, Weberschock T, Ochsendorf F. Dermatologische Lehre für Medizinstudierende: Ein Scoping Review publizierter Interventionsstudien. JDDG: J Der Deutschen Dermatologischen Gesellschaft. 2022;20(8):1077–87. 10.1111/ddg.14805_g 35971583

[24] Alvarado SM, Feng H. Representation of dark skin images of common dermatologic conditions in educational resources: a cross-sectional analysis. J Am Acad Dermatol. 2021;84(5):1427–31. 32565205 10.1016/j.jaad.2020.06.041

[25] Abdel Meguid E, Collins M. Students’ perceptions of lecturing approaches: traditional versus interactive teaching. Adv Med Educ Pract. 2017;8:229–41. 28360541 10.2147/AMEP.S131851 PMC5364003

[26] Kaur D, Singh J, Seema MA, Kaur G. Role of interactive teaching in medical education. Int J Basic Appl Med Sci. 2011;1(1):54–60.

[27] Nasmith L, Steinert Y. The evaluation of a workshop to promote interactive lecturing. Teach Learn Med. 2001;13(1):43–8. 11273379 10.1207/S15328015TLM1301_8

[28] D’Eon MF. The overcrowded curriculum is alarming. Can Med Educ J. 2023;14(4):1–5. 10.36834/cmej.78084 PMC10500406 37719410

